# Exploring the interplay of information relevance and colorfulness in multimedia learning

**DOI:** 10.3389/fpsyg.2024.1393113

**Published:** 2024-11-29

**Authors:** Juliette C. Désiron, Sascha Schneider

**Affiliations:** Institute of Education, University of Zürich, Zurich, Switzerland

**Keywords:** emotional design, information relevance, learning performance, colorfulness, decorative elements, formal learning, cognitive load

## Abstract

**Introduction:**

Looking at recent developments in multimedia learning research, the interaction between cognitive and affective processes is examined more extensively. Based on the emotional design hypothesis, for example, using colors, in contrast to black and white representations, for designing learning materials can elicit positive emotions, guide attention, increase motivation, and foster learning. The attention-guiding effect of colors might not be beneficial when used in learning-irrelevant, decorative pictures. In such a case, the seductive detail effect suggests that interesting but irrelevant additions can hinder learning. Previous studies manipulated colors in the whole learning material independent of the relevance of the information. The present study aimed to examine the effect of color variations according to the relevance of the information presented.

**Methods:**

With a 2 (colorfulness of learning-relevant information background: black and white vs. colored) × 2 (colorfulness of learning-irrelevant pictorial information: black/white vs. colored) between-subject design. The main and interaction effects of colors as attention guides of multimedia learning material on the formation of hurricanes were tested with two samples (128 university students and 140 secondary school students). The first sample is university students in laboratory conditions. The second sample is students in school classes. Besides learning outcomes, perceptions of cognitive load, metacognitive judgments, and affective states are measured.

**Results:**

Results show that the interplay between information relevance and the colorfulness of representations affects learners’ cognitive processes and metacognitive and affective perceptions.

**Discussion:**

These findings have practical implications for the design of multimedia learning materials, highlighting the importance of considering the interplay between colorfulness and information relevance.

## 1 Introduction

As digital technologies become more common and tools for editing or creating multimedia documents get easier, these multimedia materials have gradually become the standard for teaching (Seaman and Seaman, 2023). Parallel to this evolution, motivation and emotion emerged as pivotal factors influencing cognitive processing during learning, leading to the development of the cognitive-affective theory of multimedia learning (Moreno, 2005). According to this theory, the selection, organization, and integration of new information into long-term memory is moderated by learners’ motivational and emotional states. As a result, the latest empirical research on how to design multimedia learning materials investigated how the pleasantness of instructional material design influences learning (e.g., Wang et al., 2023). One central design element is using colors instead of black-and-white materials (e.g., Meusel et al., 2024). However, it is not clear if colors used in learning-relevant and learning-irrelevant parts of the material affect learners’ perceptions the same. The present study first provides insight into the effect of color manipulations (colored vs. black-and-white) with decorative pictures and learning-relevant texts and pictures. By directly manipulating color in different representations, this study aims to determine to what extent the relevance of the information moderates the previously generic learning-enhancing effect of colors.

### 1.1 Colors as an emotional design feature

Emotional design has been broadly defined in the seminal study by Um et al. (2012) as a means to trigger positive emotions from learners and enhance their learning performance. The specific features manipulated to obtain such an effect usually focus on colors, shapes, and anthropomorphic traits. Empirical studies looked into these three features concurrently or individually, mainly when applied to the sole pictorial representation of the learning materials. For example, Wang et al. (2021) investigated the effect of a monochrome representation of a poem compared to a colorful version. They found that the latter fostered positive emotions, retention, and transfer. In this study, the colors implemented were not solely those defined as warm. However, little research on text-picture materials focused on manipulating colors and how these could be implemented in both media. This is particularly important when providing practice recommendations, as textbooks or other instructional text-picture resources may include colors in the pictures and the document layout (Heidig et al., 2015; Venni and Bétrancourt, 2020). Heidig et al. (2015) manipulated color harmony (i.e., complementarity, similarity, and contrast of colors) of the structuring elements of a digital multimedia document. They found that although it did not affect differential learners’ emotional states and had a minor effect on learning outcomes, it did affect their intrinsic motivation. Higher positive emotional states after learning lead to significantly higher comprehension and intrinsic motivation.

In line with research on color preferences (Gorn et al., 1997; Palmer and Schloss, 2010), emotional design recommendations are to use highly saturated and bright colors, with shades like yellow and orange, likely to evoke positive emotions in learners (Münchow et al., 2017; Plass et al., 2014). Designing learning materials with aesthetically pleasing colors and anthropomorphism has enhanced learning outcomes (Brom et al., 2018; Wong and Adesope, 2021). Results from meta-analyses on the topic also stressed that the use of positive emotion-inducing colors must align with the learning objectives. When such was not the case, the effect of aesthetically pleasant colors on learning was either null or even harmful (e.g., Heidig et al., 2015). Although the effect on metacognitive processes is also addressed (11 out of 33 samples), Brom et al. (2018) found no significant effect of overall emotional design on perceived learning. Although color manipulations do not affect the content processed, they still affect the container processed. As such, following the cognitive load theory (CLT) (Paas and Sweller, 2021; Sweller, 2011), a colorful design could, on the one hand, lead to higher extrinsic cognitive load (ECL), which results from the design of the learning material itself. On the other hand, it should not affect the intrinsic cognitive load (ICL) inherent to the complexity of the content to be learned. However, the meta-analysis from Brom et al. (2018) pointed out the lack of clear measures of cognitive load and found materials manipulated to be designed with pleasant colors to be perceived overall as less difficult. This observation was renewed in the extended meta-analysis by Wong and Adesope (2021). The later meta-analysis also pointed out that the effects of emotional design manipulations lead to higher intrinsic motivation and lower perceived difficulty with static than animated materials, which still constituted the highest number of studied materials (20 out of 25). Moreover, with rare exceptions mentioned previously, empirical research on color as an emotional design manipulation was restricted to pictures.

### 1.2 Handling irrelevant information in multimedia: the case of decorative pictures

One argument often raised against emotional design manipulations is their potential to draw attention away from the content and thus act as a seductive detail (e.g., Kienitz et al., 2023; Park et al., 2015). Indeed, drawing too much attention to irrelevant information in the learning material tends to increase extraneous cognitive load, negatively impacting learning outcomes (Fiorella and Mayer, 2021).

The insertion of irrelevant pictures as seductive details in instructional materials has been addressed in the taxonomy of text-picture functions from Carney and Levin (2002) under the decoration category. Decorational (or decorative) pictures have “little to no relationship to the text content” and are often opposed to representational (“mirror all or part of the text content”) and interpretational (“help to clarify difficult text”) pictures, which are more often used in illustrated textbooks. While representational pictures have been found to support learning (e.g., Désiron et al., 2018a,b), such is usually not the case with decorative pictures. The meta-analysis from Levin et al. (1987) on 150 units reported no beneficial effect of decorative pictures on learning outcomes. On the other hand, the review from Rey (2012), in which decorative pictures and other seductive details were included, showed that these negatively affected learning outcomes. The review also highlighted design strategies in which seductive details positively affect learning outcomes and keep their positive effect on motivation or persistence. For example, Eitel et al. (2022) found that the presence of decorative pictures in conjunction with retrieval practice questions during a learning phase led to better-delayed retention than without such pictorial seductive details. More recently, the meta-analysis from Sundararajan and Adesope (2020) confirmed an overall negative effect of seductive details on learning outcomes but also pointed out that such was only the case when the seductive details were in the audio or the text and picture format overall. There was no overall significant effect from the mere presence of a pictorial seductive detail.

In emotional design research, a study by Schneider et al. (2016) looked into the presence of decorative pictures as a trigger for emotions when varying the emotional valence of the characters in the photograph and the environment they were set in (learning vs. leisure context). Results showed that depicted positive emotional valence led to significantly better learning outcomes than pictures with negative valence, irrespective of the context. However, the study effects on cognitive load were not controlled, although previous research reported adverse effects of seductive details on it (Park et al., 2011, 2015). Moreover, the decorative pictures used in this study were irrelevant to the content to be learned, and emotional design manipulations were restricted to those irrelevant learning content. In sum, decorative pictures might not be directly helpful for learning, but through the induction of positive emotions. The question remains: What happens if relevant, such as learning text and instructional pictures, and irrelevant information, such as decorative pictures, are presented in different coloring? Can students still distinguish their relevance when color is used in both information types?

### 1.3 The interplay between information relevance and metacognition

Metacognitive skills are crucial for assessing the relevance of information. When faced with a vast amount of information, individuals rely on their metacognitive abilities to filter out irrelevant details and focus on what is pertinent. This evaluation process involves critical thinking and reflection on how the information aligns with their goals and prior knowledge (Fisher et al., 2016). Once relevant information is identified, metacognitive regulation comes into play. This includes planning how to approach a task, monitoring comprehension and progress, and adjusting strategies as needed. For example, students might spend more time studying sections of a textbook they deem more relevant to an upcoming exam (Winne, 2018). Effective metacognition ensures that relevant information is identified and effectively utilized. This involves integrating new information with existing knowledge and applying it to solve problems or make decisions. Individuals learning with metacognitive cues, such as colors differentiating relevant and irrelevant information, are better equipped to leverage relevant information, leading to improved performance and learning outcomes (Dunlosky and Metcalfe, 2009).

### 1.4 Research questions and hypotheses

The primary aim of the present study was to investigate the interaction between the use of color as an emotional design element in multimedia documents and the relevance of the information presented. We, therefore, aimed to investigate color as an independent emotional design feature and whether it can be used to counter the effect of seductive details, here decorative pictures, on learning processes and outcomes. Based on the literature presented above, the following hypotheses were formulated:

*H1*: Based on emotional design research, instructional multimedia with a bright and colorful design should lead to higher learning outcomes, more positive emotions, higher motivation, and lower cognitive load than monochrome design.

*H2*: Following CLT, attention-grabbing design elements, such as the contrasting color of instructional parts, should lead to higher learning outcomes and lower cognitive load.

*H3*: Finally, we expect that in contrasting designs, learning should be higher and cognitive load lower, with the irrelevant information (e.g., decorative pictures) having the lowest salience.

The effect on motivation for the hypotheses is left open. As previous research on the effect of emotional design features and seductive details on the judgment of learning is scarce, we have no *a priori* hypotheses on metacognitive effects and will conduct exploratory analyses.

## 2 Methods

### 2.1 Research design, planned analyses, and power analysis

A 2 (colorfulness of learning-relevant information: black and white vs. colored) × 2 (colorfulness of decorative pictures: black and white vs. colored) between-subject design was used to examine all hypotheses. Contrasts will analyze the results based on this design and the hypotheses. The corresponding planned contrasts are presented in Table 1.

**Table 1:** Planned contrast analyses for the three hypotheses with corresponding weights.

	H1. Effect of Emotional Design	H2. Effect of Attention-grabbing	H3. The salience of seductive detail
R-BW/D-BW	−1	−1	0
R-C/D-BW	0	+1	1
R-C/D-C	+1	−1	0
R-BW/D-C	0	+1	−1

R, relevant information; D, decorative pictures; BW, black and white; C, colored.

Based on the planned contrast analysis, an *a priori* power analysis was conducted with planned contrasts (t-tests) and two tails, a moderate effect size of η*_p_*^2^ = 0.06, a test power of 1 − *β* = 0.80, and an error probability of *α* = 0.05. This analysis revealed a minimum number of participants of *N* = 128. Previous studies in emotional design were mainly conducted with university students and more scarcely with a younger population. To ensure a more significant generalization of our results, we conducted this study once with university students (see Sample 1 in section 3.4) and once with upper secondary school students (see Sample 2 in section 3.4).

### 2.2 Participants

#### 2.2.1 Participants of Sample 1

Overall, 128 university students (68.8% female; age: *M* = 22.90; *SD* = 2.84) from a German university who received either a one-hour course credit or a financial allowance were recruited. Students were enrolled in media communication (57.8%), media and instructional psychology (37.5%), and other fields of study (4.8%). Accordingly, 62.5% of the participants were bachelor students, and 37.5% were master students. Mean prior knowledge (further described in Measures) was 0.20 (SD = 0.44) out of 4 points, which can be seen as low prior knowledge. Each student was randomly assigned to one experimental group. Thirty-two participants participated in each experimental condition. In the mean, students took 625 s (*SD*: 203 s) to learn with the materials. The studies were conducted in accordance with the local legislation and institutional requirements. According to the local ethics committee, this study does not affect students ethically. Students had to give informed consent for participation.

#### 2.2.2 Participants of Sample 2

Overall, 140 school students (54.3% female, 37.1% male, 8.6% divers; age: *M* = 18.01; *SD* = 0.99) from a German secondary school. Students belonged to class 11 (40.7%), 12 (32.9%), and 13 and majored in economics (57.8%), design and media technologies (37.5%), or health and social affairs. Mean prior knowledge (further described in Measures) was 0.19 (*SD* = 0.43) out of 4 points, which can be seen as low prior knowledge. Each student was randomly assigned to one experimental group. Thirty-five participants participated in each experimental condition. In the mean, students took 652 s (*SD* = 124 s) to learn with the materials. The studies were conducted in accordance with the local legislation and institutional requirements. According to the local ethics committee, this study does not affect students ethically. Students had to give informed consent for participation.

### 2.3 Materials

#### 2.3.1 Learning materials and manipulation

**Figure 1:**
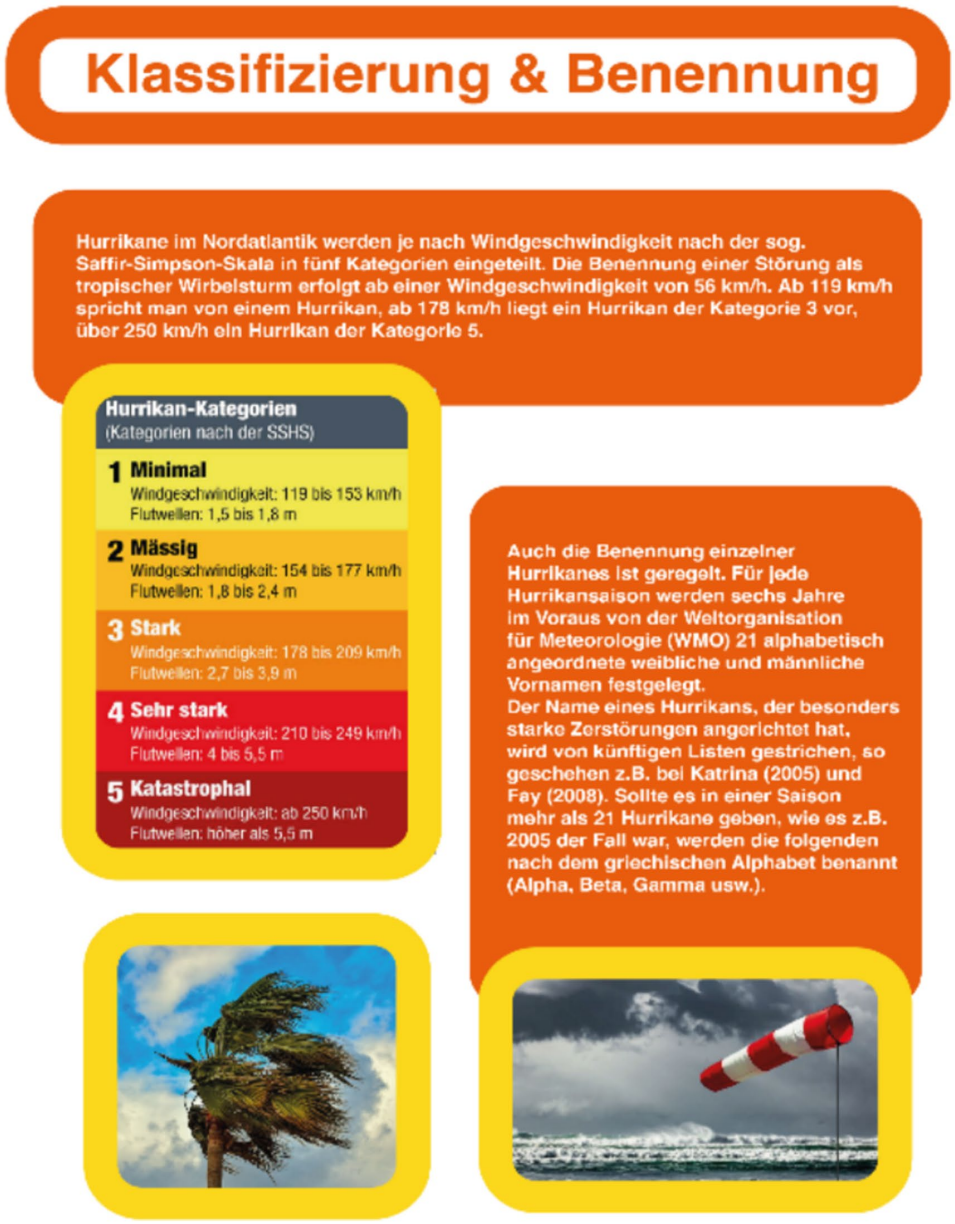
Example web page.

Four web pages on hurricanes’ structure, distribution, formation, and classification were created as learning material. These web pages consist of learning texts (820 words) and one colored instructional picture per page. In addition, colored decorative pictures were included, displaying satellite, infrared, or photographed pictures of hurricanes or causes of hurricanes (e.g., wind booms or hurricane signs). The decorative pictures were the sole irrelevant information in the learning material, with one or two included on each web page. The web pages were designed to have an aesthetically pleasing appearance (as in the study of Münchow et al., 2017), with white text on an orange background, which itself is displayed with rounded corners. In addition, headings (except for the main heading) were also displayed in colored boxes but with orange ink on a white background with an orange frame, and images were displayed with a yellow border and rounded corners (for an example web page, see Figure 1).

**Figure 2:**
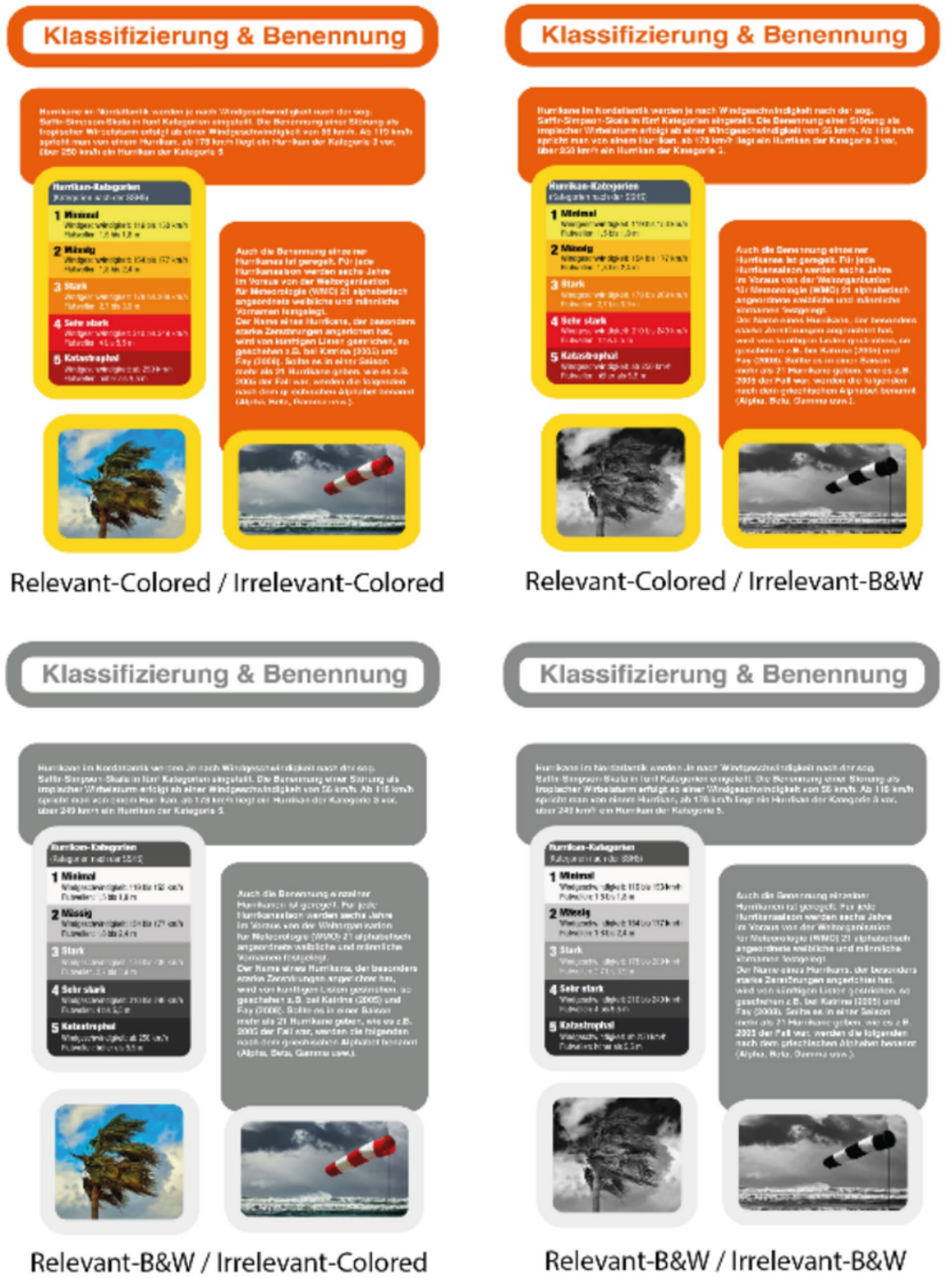
Manipulation example of a web page.

The web pages were manipulated in their colorfulness to operationalize emotional design. For non-colored conditions, all orange colors were replaced by grey with the same contrast to white to ensure no differences between the readability of the texts in both conditions. All yellow frames were exchanged for light grey frames so that the contrast between the text and heading boxes and the picture frames was the same as between the orange boxes and yellow frames in the colored versions. In addition, colored pictures were exchanged for black-and-white pictures. In versions with emotional design for relevant information, only text boxes, headings, and instructionally relevant pictures were colored. In versions with emotional design for decorative pictures, those were colored (for a manipulation example within all four experimental groups, see Figure 2).

#### 2.3.2 Measures

##### 2.3.2.1 Prior knowledge

Since the domain-specific knowledge of the learners considerably influences cognitive load perceptions and learners’ performance (Chen et al., 2017), prior knowledge was measured with one open-format question (“Which components of a hurricane can you name? “). A list with correct answers was prepared (maximum of 4 points), and participants’ answers were corrected independently by two raters (McHugh, 2012) (Sample 1: *κ* = 1.00; Sample 2: κ = 1.00).

##### 2.3.2.2 Learning outcomes: retention and transfer

Two tests (retention and transfer) were conducted to assess participants’ learning performance. Four multiple-choice and four open-recall questions were created for retention, which is defined as remembering (Mayer, 2019). Four predefined answer options could be marked as correct for each multiple-choice question. Either one, two, three, or all four items were correct. Each correctly marked answer option and each correctly unmarked answer option (for false options) was awarded one point. For example, the question “What is the connection between hurricanes and El Niño?” was provided, including the answers options: “(a) Fewer hurricanes occurred in the El Niño years,” “(b) The hurricanes were strengthened by El Niño,” “(c) There is above-average rainfall,” and “(d) The more hurricanes that occurred, the weaker the El Niño became.” For open recall questions, learners had to fill in terms in the gaps of different pictures. For example, a cross-section of a hurricane is shown as a picture, and the areas “eye of the storm,” “condensation,” “evaporation,” and “precipitation” had to be filled in. Each answer was rated by two raters (Sample 1: *κ* = [0.897, 1.000]; Sample 2: κ = [0.890, 0.965]), and one point was awarded per correct answer. Overall, for the retention test, 4 points per multiple-choice question and open-recall question could be achieved, in sum a maximum of 32 points in the retention test (Sample 1: *α* = 0.76; Sample 2: α = 0.77). For the transfer test, in which the students have to prove that they can apply the acquired knowledge (Mayer, 2019), four open-answer questions had to be answered (e.g., “Why are there no tropical storms at the equator?”). Each answer was rated by two raters (Sample 1: κ = 1.00; Sample 2: κ = 1.00), and a maximum of 4 points could be achieved per question. In conclusion, students could reach a maximum of 16 points in the transfer test. Descriptive analysis showed a sufficient difficulty in both experiments for retention (19 to 96% correct) and transfer (0 to 63% correct).

##### 2.3.2.3 Judgment of learning

Participants were prompted to anticipate their performance in a subsequent learning test, indicating their comprehension of the learning material. To achieve this, participants were presented with the following question, adapted from Son and Metcalfe (2005): “Your task will be to give a percentage from 0 to 100 of how much you think you can reproduce in a learning test what you have just learned.” This inquiry is commonly administered after the learning phase to gauge learners’ estimations of their ability to reproduce learned material in a test (Salas et al., 2011).

##### 2.3.2.4 Cognitive load

Cognitive load during learning was measured with two scales by Klepsch et al. (2017): Intrinsic cognitive load (three items, Sample 1: *α* = 0.67; Sample 2: *α* = 0.90; e.g., “This task was very complex”), and extraneous cognitive load (three items, Sample 1: *α* = 0.85; Sample 2: α = 0.92, e.g., “During this task, it was exhausting to find the important information”). Items were rated on a 10-point scale ranging from “do not agree at all” to “totally agree.”

##### 2.3.2.5 Motivation

Motivation is measured with the intrinsic motivation scale from the Situational Motivation Questionnaire by Guay et al. (2000; Sample 1: α = 0.88; Sample 2: α = 0.87). Students had to rate their motivation by providing statements related to the question: “Why are you currently engaged in this activity?” (e.g., intrinsic motivation: “Because I think that this activity is interesting”). Items were rated on a 7-point Likert scale ranging from “do not agree at all” to “totally agree.”

##### 2.3.2.6 Valence

Valence was measured using a scale from the PANAVA short scale (PANAVA-SS) by Schallberger (2005). The PANAVA-SS is a bipolar instrument that measures the valence dimension using two items: “satisfied-dissatisfied” and “unhappy-happy” (reversed) on a 7-point scale according to the question “How do you feel at the moment.” Since participants already brought an emotional state to the experiment, valence was measured before (Sample 1: α = 0.73; Sample 2: α = 0.72) and after (Sample 1: α = 0.84; Sample 2: α = 0.79) the learning material.

### 2.4 Procedure

The experiment can be separated into three phases: An *a priori* survey, a learning phase, and a *post hoc* second survey. Data on participants’ demographics, prior knowledge, and *a priori* valence were collected during the *a priori* survey. During the learning phase, participants could independently read all information on the four web pages. During the *post hoc* survey, data on participants’ *post hoc* valence, judgments of learning, motivation, cognitive load, and learning scores were collected, respectively.

## 3 Results—Sample 1

### 3.1 Preliminary analyses

Preliminary analyses of demographic data (chi-square tests) revealed no significant difference between groups (study program, *p* = 0.247). There were no significant differences for analyses of group differences (one-way ANOVA) on prior knowledge (*p* = 0.439) or *a priori* valence (*p* = 0.617). Descriptive analyses for the prerequisite variables can be found in Table 2.

**Table 2:** Means and standard deviations (in parenthesis) of prerequisite variables—Sample 1.

	Relevant information colored		Relevant information black and white	
	Decorative picture colored (*n* = 32)	Decorative picture black and white (*n* = 32)	Decorative picture colored (*n* = 32)	Decorative picture black and white (*n* = 32)
Prior knowledge	0.25 (0.57)	0.47 (0.88)	0.31 (0.47)	0.37 (0.71)
*A priori* valence	5.08 (1.00)	5.17 (0.82)	5.01 (1.10)	5.33 (1.03)

### 3.2 Learning outcome

Planned contrasts to test our first hypothesis revealed that a colorful design did not significantly affect retention (*p* = 0.926) or transfer (*p* = 0.738) compared to a monochrome design (Table 3).

**Table 3:** Means and standard deviations (in parenthesis) for learning outcomes—Sample 1.

	Relevant information colored		Relevant information black and white	
	Decorative picture colored	Decorative picture black and white	Decorative picture colored	Decorative picture black and white
Retention	17.51 (4.07)	21.39 (4.14)	20.83 (5.66)	17.62 (4.86)
Transfer	3.11 (1.07)	3.64 (1.10)	3.86 (1.14)	3.20 (1.14)

For our second hypothesis, planned contrasts revealed that a contrasted design between relevant information and decorative pictures significantly increased retention, *t*(124) = 4.23, *p* < 0.001, and transfer, *t*(124) = 3.01, *p* = 0.003, compared to having a uniform design of instructional parts.

The planned contrast for our third hypothesis was insignificant for retention (*p* = 0.635) and transfer (*p* = 0.435). The salience of the seductive detail did not affect learning outcomes.

### 3.3 Valence

Planned contrasts for *post hoc* valence showed that a colorful design was associated with significantly higher positive emotions for learners than a monochrome design, *t*(124) = 1.39, *p* < 0.001 (Table 4).

**Table 4:** Means and standard deviations (in parenthesis) for *post hoc* valence—Sample 1.

	Relevant information colored		Relevant information black and white	
	Decorative picture colored	Decorative picture black and white	Decorative picture colored	Decorative picture black and white
Valence	5.33 (0.70)	5.17 (0.70)	5.06 (0.94)	3.94 (1.30)

A contrast in the design between relevant information and decorative pictures also significantly positively affected valence, *t*(124) = 0.97, *p* = 0.004.

However, the salience of decorative pictures did not have an additional significant effect on learners’ emotions (*p* = 0.644).

### 3.4 Judgment of learning

Planned contrasts were conducted to assess the impact of emotional design. Results indicated that a colorful design did not significantly affect retention bias (*p* = 0.969), transfer bias (*p* = 0.839), or transfer accuracy (*p* = 0.319) but significantly negatively affected retention accuracy, *t*(124) = −2.771, *p* = 0.006, in comparison to a monochrome design. Retention accuracy was lower with a colorful design than with a monochrome one (Table 5).

**Table 5:** Means and standard deviations (in parenthesis) for judgment of learning on retention and transfer—Sample 1.

	Relevant information colored		Relevant information black and white	
	Decorative picture colored	Decorative picture black and white	Decorative picture colored	Decorative picture black and white
Retention bias	−4.70 (18.28)	−18.94 (14.47)	−12.59 (16.96)	−4.51 (26.08)
Retention accuracy	14.45 (11.89)	19.64 (13.47)	15.74 (13.98)	23.33 (11.81)
Transfer bias	11.16 (13.62)	2.40 (6.52)	4.26 (5.47)	10.52 (19.36)
Transfer accuracy	13.37 (11.39)	5.99 (3.37)	5.85 (3.64)	15.82 (15.19)

In terms of the grabbing attention effect, planned contrasts demonstrated that a contrasted design between relevant information and decorative pictures significantly impacted retention bias *t*(124) = −3.246, *p* = 0.002, transfer bias *t*(124) = −3.380, *p* < 0.001 or transfer accuracy *t*(124) = −4.998, *p* < 0.001, compared to a uniform design of instructional parts. Retention bias was lower with contrast than without, and a similar pattern was observed for transfer bias and accuracy. There was no significant effect on retention accuracy (*p* = 0.598).

For planned contrasts concerning the saliency of seductive details, no significant differences were observed for retention bias (*p* = 0.194), retention accuracy (*p* = 226), transfer bias (*p* = 0.555), or transfer accuracy (*p* = 0.954).

### 3.5 Cognitive load

Planned contrasts to test the effect of emotional design showed that having a colorful design did not significantly affect ICL (*p* = 1.000) or ECL (*p* = 0.430) compared to a monochrome design (Table 6).

**Table 6:** Means and standard deviations (in parenthesis) for cognitive load—Sample 1.

	Relevant information colored		Relevant information black and white	
	Decorative picture colored	Decorative picture black and white	Decorative picture colored	Decorative picture black and white
ICL	7.09 (1.35)	6.68 (1.15)	6.79 (1.53)	7.09 (1.53)
ECL	5.47(1.86)	3.88 (1.43)	3.81 (1.61)	5.16 (1.37)

Regarding the grabbing attention effect, planned contrasts revealed that a contrasted design between relevant information and decorative pictures did not significantly affect ICL (*p* = 0.430). However, they significantly decreased ECL, *t*(124) = −5.24, *p* < 0.001, compared to having a uniform design of instructional parts.

There were no significant differences for planned contrast on the saliency of seductive details for ICL (*p* = 0.744) nor ECL (*p* = 0.854).

### 3.6 Intrinsic motivation

The planned contrast for the emotional design hypothesis revealed that having a colorful design significantly improved intrinsic motivation, *t*(124) = 3.76, *p* < 0.001, compared to having a monochrome design (Table 7).

**Table 7:** Means and standard deviations (in parenthesis) of intrinsic motivation—Sample 1.

	Relevant information colored		Relevant information black and white	
	Decorative picture colored (*n* = 35)	Decorative picture black and white (*n* = 35)	Decorative picture colored (*n* = 35)	Decorative picture black and white (*n* = 35)
Intrinsic motivation	4.96 (1.03)	4.65 (0.90)	4.26 (1.18)	3.99 (0.97)

The planned contrast for the attention-grabbing effect of seductive details revealed that a contrasted design between relevant information and decorative pictures did not significantly affect intrinsic motivation (*p* = 0.898) compared to having a uniform design of instructional parts.

The planned contrast testing the saliency of the seductive details hypothesis was insignificant for intrinsic motivation (*p* = 0.292).

## 4 Results—Sample 2

### 4.1 Preliminary analyses

Preliminary analyses (chi-square tests) revealed no significant differences between groups for the year of study (*p* = 0.980). There were also no significant group differences (one-way ANOVA) for prior knowledge (*p* = 0.187) or on *a priori* valence (*p* = 0.755). Descriptive analyses for the prerequisite variables can be found in Table 8.

**Table 8:** Means and standard deviations (in parenthesis) of prerequisite variables—Sample 2.

	Relevant information colored		Relevant information black and white	
	Decorative picture colored	Decorative picture black and white	Decorative picture colored	Decorative picture black and white
Prior knowledge	0.14 (0.55)	0.51 (1.09)	0.20 (0.63)	0.43 (0.92)
*A priori* valence	4.96 (0.96)	5.00 (0.86)	4.90 (1.04)	4.76 (1.09)

### 4.2 Learning outcome

Planned contrasts to test our first hypothesis revealed that a colorful design did not significantly affect retention (*p* = 0.615) or transfer (*p* = 0.802) compared to a monochrome design (Table 9).

**Table 9:** Means and standard deviations (in parenthesis) for learning outcomes—Sample 2.

	Relevant information colored		Relevant information black and white	
	Decorative picture colored	Decorative picture black and white	Decorative picture colored	Decorative picture black and white
Retention	16.64 (4.90)	21.67 (3.84)	20.98 (4.78)	17.20 (4.90)
Transfer	2.93 (1.21)	3.70 (1.09)	3.86 (1.09)	3.00 (1.34)

For our second hypothesis, planned contrasts revealed that a contrasted design between relevant and irrelevant instructional parts significantly increased retention, *t*(136) = 5.63, *p* < 0.001, and transfer, *t*(136) = 4.05, *p* < 0.001, compared to having a uniform design of instructional parts.

The planned contrast for our third hypothesis was insignificant for retention (*p* = 0.545) and transfer (*p* = 0.581). The salience of the seductive detail did not affect learning outcomes.

### 4.3 Valence

Contrast analyses revealed that learners experienced notably higher positive emotions when exposed to a colorful design than a monochrome one, with *t*(136) = 6.97, *p* < 0.001 (Table 10).

**Table 10:** Means and standard deviations (in parenthesis) for *post hoc* valence—Sample 2.

	Relevant information colored		Relevant information black and white	
	Decorative picture colored	Decorative picture black and white	Decorative picture colored	Decorative picture black and white
Valence	4.97 (0.80)	4.83 (0.74)	4.67 (0.83)	3.54 (1.03)

Contrasting relevant and irrelevant elements in instructional materials significantly enhanced valence, as indicated by *t*(136) = 3.40, *p* < 0.001.

However, the salience of seductive details did not significantly impact learners’ emotions (*p* = 0.445).

### 4.4 Judgment of learning

Planned contrasts were conducted to assess the impact of emotional design. Results indicated that compared to a monochrome design, a colorful design did not significantly affect retention bias (*p* = 0.727) or transfer bias (*p* = 0.442) but significantly negatively affected retention bias, *t*(136) = −2.463, *p* = 0.015 and or transfer accuracy, *t*(136) = −4.486, *p* = 0.026. Retention and transfer accuracy were lower with a colorful design than a monochrome one (Table 11).

**Table 11:** Means and standard deviations (in parenthesis) for judgment of learning on retention and transfer—Sample 2.

	Relevant information colored		Relevant information black and white	
	Decorative picture colored	Decorative picture black and white	Decorative picture colored	Decorative picture black and white
Retention bias	−3.52 (17.98)	−8.71 (21.24)	−2.24 (19.66)	−1.75 (25.33)
Retention accuracy	14.24 (11.28)	18.45 (13.37)	14.92 (12.74)	21.64 (12.75)
Transfer bias	11.88 (13.65)	4.98 (10.16)	7.36 (8.32)	14.50 (21.24)
Transfer accuracy	14.28 (11.03)	9.09 (6.61)	8.96 (6.50)	20.06 (15.92)

Concerning the grabbing attention effect, planned contrasts demonstrated that a contrasted design between relevant and irrelevant instructional parts did significantly impact transfer bias *t*(136) = −2.219, *p* < 0.004 or transfer accuracy *t*(136) = −4.486, *p* < 0.001, compared to a uniform design of instructional parts. Transfer bias and accuracy were lower with contrast than without. There was no significant effect of the materials’ design on retention accuracy (*p* = 0.598) or retention accuracy (*p* = 0.556).

For planned contrasts concerning the saliency of seductive details, no significant differences were observed for retention bias (*p* = 0.205), retention accuracy (*p* = 242), transfer bias (*p* = 0.486), or transfer accuracy (*p* = 0.958).

### 4.5 Cognitive load

Planned contrasts to test the effect of emotional design showed that a colorful design did not significantly affect ICL (*p* = 0.937) or ECL (*p* = 0.940) compared to a monochrome design (Table 12).

**Table 12:** Means and standard deviations (in parenthesis) for cognitive load—Sample 2.

	Relevant information colored		Relevant information black and white	
	Decorative picture colored	Decorative picture black and white	Decorative picture colored	Decorative picture black and white
ICL	7.25 (1.50)	6.98 (1.30)	6.68 (1.82)	7.28 (1.35)
ECL	5.75 (1.71)	4.48 (1.50)	4.36 (1.72)	5.78 (1.36)

Regarding the grabbing attention effect, planned contrasts revealed that a contrasted design between relevant and irrelevant instructional parts did not significantly affect ICL (*p* = 0.095). However, they significantly decreased ECL, *t*(136) = −5.04, *p* < 0.001, compared to having a uniform design of instructional parts.

There were no significant differences in planned contrast on the saliency of seductive details for ICL (*p* = 0.415) or ECL (*p* = 0.763).

### 4.6 Intrinsic motivation

The planned contrast for the emotional design hypothesis revealed that having a colorful design significantly improved intrinsic motivation, *t*(136) = 3.62, *p* < 0.001, compared to having a monochrome design (Table 13).

**Table 13:** Means and standard deviations (in parenthesis) of intrinsic motivation—Sample 2.

`	Relevant information colored		Relevant information black and white	
	Decorative picture colored	Decorative picture black and white	Decorative picture colored	Decorative picture black and white
Intrinsic motivation	4.90 (1.05)	4.61 (0.99)	4.20 (1.14)	4.00 (0.96)

The planned contrast for the attention-grabbing effect of seductive details revealed that a contrasted design between relevant and irrelevant instructional parts did not significantly affect intrinsic motivation (*p* = 0.792) compared to having a uniform design of instructional parts.

The saliency of the seductive detail hypothesis planned contrast was not significant for intrinsic motivation (*p* = 0.104).

## 5 Discussion

This study explored how using color to differentiate relevant and irrelevant information in multimedia learning materials impacts learners’ cognitive load, emotional responses, and metacognitive processes. Our findings contribute to a deeper understanding of the interplay between emotional and cognitive factors in learning, expanding on the cognitive-affective theory of multimedia learning (Moreno, 2005) and providing practical guidance for instructional design.

### 5.1 Key findings

Two key findings from our results should be highlighted. First, the use of color to contrast relevant and irrelevant information was shown to improve learners’ ability to identify essential content, leading to better performance in retention and transfer tasks. This supports the idea that visual design elements, such as color, are crucial in guiding attention and organizing information in ways that facilitate learning. Our results thus resonate with the CATLM, emphasizing that learners’ emotional and cognitive states jointly influence their ability to process and retain information (Moreno, 2005). The findings are particularly relevant in light of previous research on emotional design, which has shown that colors can evoke positive emotions and enhance motivation (Um et al., 2012; Brom et al., 2018). They also point out the fine line in the use of color as an emotional trigger as well as a means to signal to learners where to direct their attention, as highlighted by research on the signaling principle in text-picture documents (e.g., Beege et al., 2021; Désiron et al., 2018a,b, 2021).

Second, color contrast reduced ECL, suggesting learners could better manage the materials’ complexity and focus on the key information. However, while ECL decreased, ICL was not significantly affected, implying that the inherent difficulty of the learning content remained unchanged (Sweller, 2011). By demonstrating that color contrast reduces ECL, our study supports the idea that carefully designed multimedia materials can ease cognitive processing demands. This aligns with Sweller (2011) CLT, highlighting the importance of reducing unnecessary cognitive effort to enhance learning outcomes.

The study also found that color contrast positively influenced learners’ judgment of their learning, a critical metacognitive skill that allows learners to monitor and adjust their study strategies (Fisher et al., 2016). These findings align with the broader literature on emotional design, which posits that aesthetically engaging materials can enhance emotional engagement and cognitive processing (Um et al., 2012; Plass et al., 2014). However, our results extend this work by highlighting the specific role of color in distinguishing relevant from irrelevant content, thereby optimizing cognitive and metacognitive processes.

Interestingly, our study challenges the seductive detail effect (Sundararajan and Adesope, 2020), which suggests that decorative elements irrelevant to the learning content may hinder learning by increasing cognitive load. In contrast, we found that the salience of seductive details—when differentiated by color—did not have the detrimental effects often reported in previous research (Rey, 2012; Sundararajan and Adesope, 2020). This divergence suggests that seductive details might be less harmful when visually distinct from relevant information, allowing learners to easily filter out distractions and focus on the essential content. This finding calls for further investigation into how color and other design elements can mitigate the negative impact of irrelevant information in learning environments.

### 5.2 Color contrast and cognitive load

Our study provides compelling evidence that color contrast can significantly reduce ECL by helping learners better organize and process information. The reduction in ECL suggests that learners could more easily differentiate between essential and non-essential content, thus minimizing the effort required to navigate through irrelevant information. This finding is significant in CLT, which posits that effective instructional design should aim to reduce extraneous load to free up cognitive resources for deeper learning processes (Paas and Sweller, 2021).

Moreover, our results indicate that while color contrast reduces ECL, it does not significantly affect ICL. This suggests that the inherent difficulty of the content remains unchanged, and learners must still engage in complex cognitive processes to understand the material. This finding highlights the nuanced role of emotional design. While color can help streamline cognitive processes by reducing unnecessary distractions, it does not fundamentally alter the cognitive effort required to comprehend the core content. This distinction between extraneous and ICL is critical for instructional designers, as it suggests that color should be used to support learners’ cognitive efficiency without oversimplifying or diluting the complexity of the learning material.

### 5.3 Emotional and cognitive effects of color

A key question of this study was examining how color affects both emotional and cognitive dimensions of learning. Our findings confirm that using color can evoke positive emotional responses, enhancing learners’ motivation and engagement with the material. This supports the emotional design hypothesis, posing that aesthetically pleasing design elements can create a more enjoyable and motivating learning experience (Plass et al., 2014). In particular, learners exposed to color contrasts reported higher levels of intrinsic motivation and positive valence, which aligns with prior research indicating that emotionally engaging materials can foster deeper cognitive engagement (Wang et al., 2021).

Importantly, the interaction between emotional and cognitive effects was evident in how color influenced metacognitive judgments. Learners with color contrasts could better assess their learning, making more accurate judgments about their comprehension and retention of the material. This suggests that color enhances the emotional appeal of learning materials and improves metacognitive accuracy by helping learners better gauge their understanding of the content. These findings align with the CATML assumption (Moreno and Mayer, 2007) and underscore the need for a holistic approach to instructional design, where emotional and cognitive factors are considered to optimize learning outcomes.

### 5.4 Practical implications

The findings of this study have several practical implications for instructional designers and educators. First, using color contrast to distinguish relevant from irrelevant information can effectively reduce cognitive load and improve learning outcomes. By helping learners focus on the essential content, color contrast enhances comprehension and improves learners’ ability to assess their performance and make accurate judgments of learning.

Second, the positive emotional effects of color underscore the importance of considering aesthetic design elements in educational materials. While color alone may not reduce the perceived ICL of the material, it can enhance learners’ motivation and engagement, leading to a more enjoyable and productive learning experience. Therefore, instructional designers should create visually appealing materials in which color is used strategically to support cognitive and emotional learning aspects.

Finally, our findings suggest that seductive details may not be included when differentiated from relevant content. This has important implications for the design of multimedia materials, particularly in cases where aesthetically pleasing but non-essential elements are used to enhance engagement. Instructional designers should ensure that such elements are visually distinct from the learning content. This allows learners only to pay limited attention to distractions and focus on what is most important.

### 5.5 Theoretical implications

One of the most unexpected findings of this study was the lack of a significant effect of color on ICL. While we hypothesized that color would facilitate cognitive processing across the board, the results suggest that color’s impact is primarily on ECL rather than the core difficulty of the material (i.e., on ICL). This finding challenges the assumption that emotional design elements like color can directly influence the intrinsic demands of a learning task (Plass and Kalyuga, 2019). Instead, our results indicate that color plays a more supportive role, aiding learners in organizing and navigating the material but not necessarily making the content more accessible.

The study also contributes to the CATML (Moreno and Mayer, 2007) by demonstrating color’s specific role in enhancing cognitive and emotional processes. While previous research has focused primarily on the emotional effects of grayscale vs. warm colors (e.g., Le et al., 2021; Münchow and Bannert, 2019), our study adds to this body of work by showing that contrasting color use relative to information relevance can also improve cognitive efficiency by reducing extraneous cognitive load and improving learners’ metacognitive judgments. These findings suggest that the cognitive and emotional effects of color are closely intertwined, and both must be considered to fully understand how design elements influence learning.

Additionally, the finding that the salience of seductive details did not negatively impact learning outcomes was surprising. This contrasts with much previous research, showing that seductive details can increase cognitive load and detract from learning (Rey, 2012). Our results suggest that when irrelevant information is differentiated from relevant content—such as through color contrast—learners can better filter out distractions and focus on the critical material. This has important implications for the design of multimedia materials, aligning with the idea of Eitel et al. (2019, 2022), as it suggests that seductive details may not be inherently harmful if they are visually distinct and do not compete for learners’ attention. This invites further exploration of how different design elements, such as color, shape, and layout, can guide attention and support learning without increasing cognitive load.

### 5.6 Limitations and future directions

As with any scientific study, the experiment conducted to investigate the effects of color and emotional design in learning materials is not without its limitations. Recognizing these limitations is crucial for comprehensively understanding the research findings and informing future directions in this field.

One limitation of the study lies in the manipulation of irrelevant information, which was solely accomplished through the inclusion of pictures. Future research should explore how irrelevant verbal information impacts learning outcomes, as this may yield different results and provide a more nuanced understanding of the role of irrelevant stimuli in educational materials. While the study focused on the emotional impact of color in learning materials, emotional design encompasses a broader range of features, including anthropomorphism, shapes, and aesthetics. Investigating these additional dimensions of emotional design is essential for generalizing the findings and elucidating the elements contributing to enhanced learning outcomes. The manipulations employed in the study may have influenced dimensions of motivation or affect beyond intrinsic motivation. Future research should explore how variations in design elements affect other aspects of motivation, such as interest and activation, to provide a more comprehensive understanding of the emotional and motivational dynamics at play in learning environments.

Although the study examined the effects of color on learning outcomes and motivation, incorporating eye-tracking data could offer valuable insights into the attention-grabbing effects of colors. Analyzing participants’ gaze patterns could provide a more detailed understanding of how color influences attention allocation and information processing during learning tasks. The study utilized only two colors, which may limit the generalizability of the findings to all colors. To establish more robust conclusions regarding the effects of color on learning, future research should explore a broader spectrum of colors and examine how hue, saturation, and brightness variations impact learning outcomes and motivation. In some instances, the study found that the manipulations did not significantly affect learning outcomes. This could be attributed to limitations in the way learning outcomes were measured. Future research should employ diverse assessment tools and methodologies to capture the full spectrum of learning outcomes, including comprehension, retention, and knowledge transfer. Also, future studies should investigate how mediating variables can explain the effects found in the experiments. In particular, the relationship between color, emotion, and metacognitive judgments needs to be examined further (for a review, see Clore and Huntsinger, 2007). This study is also limited by its sample diversity, reliance on self-report measures, short intervention time, and a more extensive prior knowledge measurement (e.g., a pre-post-test design). According to the non-significant results, future studies can also test with a Bayesian approach for null effects. Since the findings on cognitive load are inconsistent, and the positive motivational impacts do not translate to better learning outcomes, further exploration is needed. A more nuanced measurement of emotions and its impact on motivation can increase the explainability of the results.

In conclusion, while the study provides valuable insights into the effects of color and emotional design on learning outcomes and motivation, it is essential to acknowledge and address its limitations. By addressing these limitations and pursuing future research, we can advance our understanding of how design elements influence learning processes and develop more effective educational interventions.

## 6 Conclusion

In conclusion, this study provides valuable insights into how color contrast between relevant and irrelevant information can reduce cognitive load, enhance metacognitive judgments, and foster positive emotional engagement in multimedia learning environments. These findings highlight the importance of integrating cognitive and emotional considerations into instructional design, ensuring learners are cognitively supported and emotionally engaged. Educators and instructional designers can create more effective and enjoyable learning experiences that optimize cognitive and emotional processes by strategically using color and other design elements. Future research should continue to explore how different aspects of emotional design influence learning outcomes, focusing on the interplay between cognitive load, motivation, and metacognitive accuracy.

## Data Availability

The raw data supporting the conclusions of this article will be made available by the authors, without undue reservation.
